# The Unique Maximal *GF*-Regular Submodule of a Module

**DOI:** 10.1155/2013/750808

**Published:** 2013-09-15

**Authors:** Areej M. Abduldaim, Sheng Chen

**Affiliations:** Department of Mathematics, Harbin Institute of Technology, Harbin 150001, China

## Abstract

An *R*-module *A* is called *GF*-regular if, for each *a* ∈ *A* and *r* ∈ *R*, there exist *t* ∈ *R* and a positive integer *n* such that *r* ^*n*^ *tr* ^*n*^ *a* = *r* ^*n*^ *a*. We proved that each unitary *R*-module *A* contains a unique maximal *GF*-regular submodule, which we denoted by *M* *GF*(*A*). Furthermore, the radical properties of *A* are investigated; we proved that if *A* is an *R*-module and *K* is a submodule of *A*, then *M* *GF*(*K*) = *K*∩*M* *GF*(*A*). Moreover, if *A* is projective, then *M* *GF*(*A*) is a *G*-pure submodule of *A* and *M* *GF*(*A*) = *M*(*R*) · *A*.

## 1. Introduction

Throughout this paper, *R* is a commutative ring with identity and all modules are left unitary, unless otherwise stated. Recall that an element *r* ∈ *R* is said to be regular if there exists *t* ∈ *R* such that *r* *tr* = *r*; a ring *R* is called regular if and only if each element of *R* is regular. An ideal *I* of a ring *R* is regular if each of its elements is regular in *R*; indeed, a regular ideal *I* of *R* is itself a regular ring [1]. Brown and McCoy proved in [1] that each ring *R* contains a unique maximal regular ideal *M*(*R*) which satisfies the well-known radical properties. The ideal *M*(*R*) is called the regular radical of *R*.

The concept of regularity was extended to modules in several ways and in [2] the notion of *F*-regular modules (in the sense of Fieldhouse [3]) was generalized to *GF*-regular modules. Let *A* be an *R*-module; an element *a* ∈ *A* is said to be *GF*-regular if for each *r* ∈ *R* there exist *t* ∈ *R* and a positive integer *n* such that *r* ^*n*^ *tr* ^*n*^ *a* = *r* ^*n*^ *a*. An *R*-module *A* is called *GF*-regular if and only if all its elements are *GF*-regular; in particular, a ring *R* is *GF*-regular if and only if *R* is *GF*-regular as an *R*-module. On the other hand a ring *R* is *π*-regular if and only if *R* is a *GF*-regular *R*-module; recall that a ring *R* is *π*-regular if, for each *r* ∈ *R*, there exist *t* ∈ *R* and a positive integer *n* such that *r* ^*n*^ *tr* ^*n*^ = *r* ^*n*^. A submodule *N* of an *R*-module *A* is called *GF*-regular if each element of *N* is *GF*-regular and every submodule of a *GF*-regular module is a *GF*-regular module. Also, in [2] the concept of *G*-pure submodules was introduced; a submodule *P* of an *R*-module *A* is called *G*-pure if, for each *r* ∈ *R*, there exists a positive integer *n* such that *P*∩*Rr* ^*n*^ *A* = *Rr* ^*n*^ *P*.

In this paper we show that each module contains a unique maximal *GF*-regular submodule, which we denote by *M* *GF*(*A*), and we show that *M* *GF*(*A*) satisfies some but not all of the usual radical properties.

## 2. Main Results

**Theorem 1**

Let *R* be any ring. Every *R*-module contains a unique maximal *GF*-regular submodule.

**Proof**

Let *R* be any ring, let *A* be an *R*-module, and let

(1) $$\begin{array}{c} G=\left\{N\;|\;N\;\text{is}\;\text{a}\;GF\text{-regular}\;\text{submodule}\;\text{of}\;A\right\}, \end{array}$$

where *G* ≠ *ϕ* because (0) is a *GF*-regular submodule of *A*. Let {*N*_*i*_} be an ascending chain in *G* and *B* = ⋃_*i*∈Λ_ *N*_*i*_. Let *b* ∈ *B*; there exists *j* ∈ Λ such that *b* ∈ *N*_*j*_, but *N*_*j*_ is a *GF*-regular submodule; then; for each *r* ∈ *R*, there exist *t* ∈ *R* and a positive integer *n* such that *r* ^*n*^ *tr* ^*n*^ *b* = *r* ^*n*^ *b*; therefore *b* is a *GF*-regular element in *B* which implies that *B* is a *GF*-regular *R*-module. Now, by Zorn's lemma, *G* contains a maximal element which we call *M* *GF*. To prove the uniqueness of *M* *GF*, assume that *M* *GF*1 and *M* *GF*2 be two maximal *GF*-regular submodules in *A*; then for any maximal ideal *P* of *R* each of *M* *GF*1_*p*_ and *M* *GF*2_*p*_ is semisimple over *R*_*p*_ [2, Proposition 21]. Now, let *M* *GF*1_*p*_∩*M* *GF*2_*p*_ = *K*_*p*_; then *K*_*p*_⊆*M* *GF*1_*p*_ and *K*_*p*_⊆*M* *GF*2_*p*_; thus *M* *GF*1_*p*_ = *K*_*p*_ + *A*1_*p*_ and *M* *GF*2_*p*_ = *K*_*p*_ + *A*2_*p*_, where *A*1_*p*_ and *A*2_*p*_ are two submodules of *A*_*p*_ [4]. Hence, *M* *GF*1_*p*_ + *M* *GF*2_*p*_ = *A*1_*p*_ + *K*_*p*_ + *A*2_*p*_, but each of *A*1_*p*_, *A*2_*p*_, and *K*_*p*_ is a semisimple submodule; thus *M* *GF*1_*p*_ + *M* *GF*2_*p*_ is a semisimple submodule which implies that *M* *GF*1_*p*_ + *M* *GF*2_*p*_ is *GF*-regular [2]. So *M* *GF*1 + *M* *GF*2 is a *GF*-regular submodule [2, Theorem 20]. Now, each of *M* *GF*1 and *M* *GF*2 is a maximal *GF*-regular submodule and hence *M* *GF*1 + *M* *GF*2 = *M* *GF*2 = *M* *GF*1.

**Remark 2**

We denote the unique maximal *GF*-regular submodule of an *R*-module *A* by *M* *GF*(*A*). It is obvious that *M* *GF*(*A*) contains every *GF*-regular submodule of *A*; this means that *M* *GF*(*A*) is a *GF*-regular submodule which is not contained properly in any other *GF*-regular submodule. In fact, *M* *GF*(*A*) is the sum of all *GF*-regular submodules of *A* and *M* *GF*(*A*) = *A* if and only if *A* is a *GF*-regular module.

**Example 3**

(a) Since the *Z*-module *Z*_*n*_ is *GF*-regular for each positive integer *n* [2], then *M* *GF*(*Z*_*n*_) = *Z*_*n*_.

(b) Each element in the *Z*-module *Q* is not *GF*-regular [2]; hence *M* *GF*(*Q*) = (0).

(c) Let *p* be a prime number and let *A* = *Z*_*p*^*∞*^_ = ⋃_∀*i*_ *Z*_*p*^*i*^_ be a *Z*-module. Let *a* ∈ ⋃_∀*i*_ *Z*_*p*^*i*^_; then there exists a positive integer *m* such that *a* ∈ *Z*_*p*^*m*^_, but *Z*_*n*_ is a *GF*-regular *Z*-module for each positive integer *n*; hence *a* is a *GF*-regular element, so *a* ∈ *M* *GF*(*Z*_*p*^*∞*^_) which implies that *M* *GF*(*Z*_*p*^*∞*^_) = *Z*_*p*^*∞*^_.

(d) Let *Q*/*Z* be a *Z*-module; since *Q*/*Z* is a torsion *Z*-module, then *Q*/*Z* is a *GF*-regular *Z*-module [2, Proposition 6]. Since *Q*/*Z* = ∑_*p*_ *Z*_*p*^*∞*^_ for each prime number *p*, then *M* *GF*(*Q*/*Z*) = ∑_*p*_ *Z*_*p*^*∞*^_ for all primes *p*.

**Proposition**

Let *A* and *B* be *R*-modules, and let *K* be a submodule of *A*; then

- *M* *GF*(*K*) = *K*∩*M* *GF*(*A*),
- *M* *GF*(*A* ⊕ *B*)⊆*M* *GF*(*A*) ⊕ *M* *GF*(*B*).

**Proof**

(a) Let *K* be a submodule of *A*, and let *k* ∈ *M* *GF*(*K*); then *k* ∈ *K* and *k* is *GF*-regular in *K* which implies that *k* is *GF*-regular in *A*, thus *k* ∈ *K*∩*M* *GF*(*A*). Conversely, let *k* ∈ *K* and *k* ∈ *M* *GF*(*A*); therefore *k* is *GF*-regular in *K* which means that *k* ∈ *M* *GF*(*K*) and hence *M* *GF*(*K*) = *K*∩*M* *GF*(*A*).

(b) Let *c* ∈ *M* *GF*(*A* ⊕ *B*); then *c* = (*a*, *b*), where *a* ∈ *A* and *b* ∈ *B*. Since *c* is *GF*-regular, then each of *a* and *b* is *GF*-regular which means that *a* ∈ *M* *GF*(*A*) and *b* ∈ *M* *GF*(*B*); hence *c* ∈ *M* *GF*(*A*) ⊕ *M* *GF*(*B*).

**Proposition 5**

Let *A* and *A*′ be *R*-modules, and let *f* : *A* → *A*′ be an *R*-homomorphism; then *f*(*M* *GF*(*A*))⊆*M* *GF*(*f*(*A*)).

**Proof**

If *a* ∈ *M* *GF*(*A*), then *R*/*ann*(*a*) is a *π*-regular ring, but *ann*(*a*)⊆*ann*(*f*(*a*)); thus *R*/*ann*(*f*(*a*)) is an epimorphic image of *R*/*ann*(*A*); hence it is a *π*-regular ring. Therefore, *f*(*a*) ∈ *M* *GF*(*f*(*a*)) and *f*(*M* *GF*(*A*))⊆*M* *GF*(*f*(*A*)).

**Remark 6**

(a) If *f* : *A* → *A*′ is an *R*-epimorphism, then *f*(*M* *GF*(*A*)) ≠ *M* *GF*(*A*′) in general. In fact, let *π* : *Z* → *Z*_4_≃*Z*/4*Z* be the natural map, where *Z* and *Z*_4_ are *Z*-modules. It is easy to check that *M* *GF*(*Z*) = (0), *f*(*M* *GF*(*Z*)) = (0), but *M* *GF*(*Z*_4_)≃*Z*_2_.

(b) It is shown in [1] that, for a ring *R*, *M*(*R*/*M*(*R*)) = (0) which is not true in case of *GF*-regular modules; this means that *M* *GF*(*A*/*M* *GF*(*A*))≠(0) (as in (a)).

**Corollary 7**

For each *R*-module *A*, *M*(*R*) · *A*⊆*M* *GF*(*A*).

**Proof**

For each *a* ∈ *A*, let *f* : *R* → *A* be an *R*-homomorphism defined by *f*(*r*) = *ra*. Then *f*(*M*(*R*))⊆*M* *GF*(*A*) by Proposition 5, but *M*(*R*) · *A* = ∑_*a*_ *f*(*M*(*R*)); hence *M*(*R*) · *A*⊆*M* *GF*(*A*).

Let *J*(*R*) be the Jacobson radical of a ring *R*. Brown and McCoy proved in [1] that *M*(*R*)∩*J*(*R*) = (0). However, this is not true for *GF*-regular modules; for example, if *A* = *Z*_4_ is a *Z*-module, then *M* *GF*(*A*)≃*Z*_2_, *J*(*A*)≃*Z*_2_ and *M* *GF*(*A*)∩*J*(*A*)≠(0).

**Lemma 8**

Let *A* be an *R*-module and let *P* be a *G*-pure submodule of *A*. For any *r* ∈ *R*, there exists a positive integer *n* such that *P* = *Rr* ^*n*^ *P* if and only if *P*⊆*Rr* ^*n*^ *A*.

**Proof**

Since *P* is *G*-pure in *A*, then, for each *r* ∈ *R*, there exists a positive integer *n* such that *P*∩*Rr* ^*n*^ *A* = *Rr* ^*n*^ *P*. If *P* = *Rr* ^*n*^ *P*, then *P*∩*Rr* ^*n*^ *A* = *P*, and hence *P*⊆*Rr* ^*n*^ *A*. Conversely, if *P*⊆*Rr* ^*n*^ *A*, then *P*∩*Rr* ^*n*^ *A* = *P*, but *P*∩*Rr* ^*n*^ *A* = *Rr* ^*n*^ *P*; therefore *P* = *Rr* ^*n*^ *P*.

**Lemma 9**

Let *r* ∈ *J*(*R*); if *P* is a finitely generated *G*-pure submodule of an *R*-module *A* such that *P*⊆*Rr* ^*n*^ *A* for some positive integer *n*, then *P* = 0.

**Proof**

By Lemma 8 we get that *P* = *Rr* ^*n*^ *P* and by Nakayama's lemma [5], *P* = 0.

**Theorem 10**

Let *A* be an *R*-module. If *M* *GF*(*A*) is a *G*-pure submodule of *A*, then *M* *GF*(*A*)∩*J*(*R*) · *A* = (0).

**Proof**

Let *r* ∈ *M* *GF*(*A*)∩*J*(*R*) · *A*, and let *P* = *Rr*. It is clear that *P*⊆*M* *GF*(*A*). Since *M* *GF*(*A*) is a *GF*-regular module, then *P* is a *G*-pure submodule in *M* *GF*(*A*) [2, Theorem 11]. But *M* *GF*(*A*) is *G*-pure in *A*; hence *P* is *G*-pure in *A*. Now, *P*⊆*J*(*R*) · *A*, so *P* = 0 by Lemma 9. Therefore *M* *GF*(*A*)∩*J*(*R*) · *A* = (0).

Recall that *M*(*R*) is always a pure ideal in *R*. Hence *M*(*R*) is *G*-pure [2].

**Theorem 11**

Let *A* be a projective *R*-module; then

- *M* *GF*(*A*) = *M*(*R*) · *A*,
- *M* *GF*(*A*) is a *G*-pure submodule of *A*,
- *M* *GF*(*A*)∩*J*(*A*) = (0).

**Proof**

(a) By the dual basis lemma [4], for each *a* ∈ *A* we have that *a* = ∑_*i*_ *f*_*i*_(*a*)*a*_*i*_, where *a*, *a*_*i*_ ∈ *A* for all *i* and *f*_*i*_ ∈ A* : = Hom_*R*_(*A*, *R*). If *a* ∈ *M* *GF*(*A*), then the submodule *Ra* is *GF*-regular and *f*_*i*_(*Ra*) is a *GF*-regular ideal in *R* by Proposition 5, hence *M*(*R*). Thus *M* *GF*(*A*)⊆*M*(*R*) · *A*. We get the other direction of the inclusion by Corollary 7.

(b) First we claim that; for any two ideals *K* and *L* of *R*, (*K* ∩ *L*)*A* = *KA* ∩ *LA*; it is enough to show this locally; thus we may assume that *A* is free. It is clear that (*K*∩*L*)*A*⊆*KA*∩*LA*. On the other hand, let *x* ∈ *KA*∩*LA*; then

(2) $$\begin{array}{c} x=\sum r_{i}x_{i}=\sum s_{i}x_{i}\;r_{i}\in K,\;\;s_{i}\in L. \end{array}$$

By freeness, *r*_*i*_ = *s*_*i*_ and *x* ∈ (*K*∩*L*)*A*. Now, let *K* be any ideal in *R*; then by (a) we get *M* *GF*(*A*) ∩ *KA* = *M*(*R*) ∩ *KA* = (*M*(*R*) ∩ *K*)*A*. But *M*(*R*) is *G*-pure ideal, so, for each *r* ∈ *R*, there exists a positive integer *n* such that *M*(*R*)∩*Rr* ^*n*^ = *Rr* ^*n*^ *M*(*R*); hence *M* *GF*(*A*)∩*Rr* ^*n*^ *A* = *Rr* ^*n*^ *M*(*R*) · *A* = *Rr* ^*n*^ *M* *GF*(*A*).

(c) Since *A* is projective, then *J*(*A*) = *J*(*R*) · *A* [4] which implies that *M* *GF*(*A*)∩  *J*(*A*) = (0) by Theorem 10.

**Corollary 12**

Let *R* be any ring, and let *A* be any *R*-module such that *M* *GF*(*A*) is a *G*-pure submodule and *J*(*A*) = *J*(*R*) · *A*; then *M* *GF*(*A*)∩*J*(*A*) = (0).

**Proof**

Since *J*(*A*) = *J*(*R*) · *A*, then by Theorem 10 we get that *M* *GF*(*A*)∩*J*(*A*) = (0).

**Remark 13**

If *A* is a *GF*-regular *R*-module, then *M* *GF*(*A*) = *A*; hence *J*(*R*) · *A* = *J*(*R*) · *A*∩*A* = *J*(*R*) · *A*∩*M* *GF*(*A*) = (0). In fact, this shows that Theorem 10 is a generalization of [2, Proposition 28].

In [2] we noticed that every module over *π*-regular ring is *GF*-regular, but the converse need not be true in general. The next result shows how the converse may be true, but first we recall that if *A* is an *R*-module, then the trace of *A* is tr⁡(*A*) = ∑_*f*∈A*_ *f*(*A*), where A* = Hom(*A*, *R*).

**Proposition 14**

Let *A* be a *GF*-regular *R*-module. If tr⁡(*A*) = *R*, then *R* is a *π*-regular ring.

**Proof**

For each *a* ∈ *A* and *f* ∈ A* = Hom(*A*, *R*), since *Ra* is a *GF*-regular submodule of *A*, then by [2, Proposition 7] we get that *f*(*Ra*) is a *π*-regular ideal. Thus *f*(*Ra*)⊆*M*(*R*), but *f*(*Ra*)⊆tr⁡(*A*); hence tr⁡(*A*) = *R*⊆*M*(*R*), which implies that *M*(*R*) = *R* and *R* is *π*-regular.
